# New mechanisms of multidrug resistance: an introduction to the *Cancer Drug Resistance* special collection

**DOI:** 10.20517/cdr.2023.86

**Published:** 2023-08-17

**Authors:** Michael M. Gottesman, Robert W. Robey, Suresh V. Ambudkar

**Affiliations:** Laboratory of Cell Biology, Center for Cancer Research, National Cancer Institute, National Institutes of Health, Bethesda, MD 20892, USA.

**Keywords:** Cancer drug resistance, multidrug resistance, ABC transporters, novel mechanisms of drug resistance

## Abstract

*Cancer Drug Resistance* publishes contributions to understanding the biology and consequences of mechanisms that interfere with successful treatment of cancer. Since virtually all patients who die of metastatic cancer have multidrug-resistant tumors, improved treatment will require an understanding of the mechanisms of resistance to design therapies that circumvent these mechanisms, exploit these mechanisms, or inactivate these multidrug resistance mechanisms. One example of a resistance mechanism is the expression of ATP-binding cassette efflux pumps, but unfortunately, inhibition of these transporters has not proved to be the solution to overcome multidrug resistance in cancer. Other mechanisms that confer multidrug resistance, and the confluence of multiple different mechanisms (multifactorial multidrug resistance) have been identified, and it is the goal of this Special Collection to expand this catalog of potential multidrug resistance mechanisms, to explore novel ways to overcome resistance, and to present thoughtful reviews on the problem of multidrug resistance in cancer.

## WHY MULTIDRUG RESISTANCE IN CANCER REMAINS AN UNSOLVED PROBLEM

The molecular understanding of the factors that contribute to the growth, invasiveness, and metastasis of cancer has led to an explosion of new treatments for cancer, including the development of targeted chemotherapeutics. Unfortunately, although there have been clear improvements in early diagnosis and treatment of cancer, leading to improved prognosis and duration of survival, the patient with a metastatic solid tumor or many kinds of hematologic cancer will not be cured and will eventually succumb to their disease. This leads inevitably to the conclusion that curative treatments for cancer will require a much better understanding of the many mechanisms that result in resistance to treatment, especially multidrug resistance to chemotherapy.

A summary of the various means by which cancer cells might resist chemotherapy was published in 2016^[1]^. These categories are still relevant today and include: (1) For targeted drugs, alterations by mutation in the targets or activation of alternative growth promoting pathways; (2) Changes in cell-based pharmacology, including drug efflux pumps, altered uptake systems, altered metabolism, sequestration and altered trafficking of drugs, *etc*.; (3) Epigenetic alterations in differentiation pathways, such as Epithelial to Mesenchymal Transition (EMT), altered transcription patterns, cell survival adaptations, loss of cell death pathways and stem cell-like states; and (4) Alterations in local tumor physiology and substratum, including other tumor-associated cells, changes in physical properties of the substratum, and altered blood supply. All of these potential resistance mechanisms can be facilitated by many of the known hallmarks of cancer, such as heterogeneity and genetic and epigenetic plasticity, which now include several characteristics such as resistance to apoptosis and avoidance of immune destruction, which help confer resistance to anti-cancer drugs^[2]^.

Recent reviews and original research articles have identified some novel mechanisms of resistance that fall into these categories but were unexplored until recently. These include the concepts of dormancy^[3]^, persistence^[4]^, metabolic plasticity^[5]^, the role of tumor-associated fibroblasts^[6]^, the integrated stress response^[7]^, the role of long noncoding RNAs (lncRNAs)^[8]^, microvesicles^[9]^, and transcriptional variability^[10]^. To no one’s surprise, the microbiome has turned out to affect response to chemotherapy^[11]^ and the constituents and physical properties of the extracellular matrix are also determinative^[12]^. In addition, the use of machine deep learning to analyze the response of human cancer cells to therapy has been proposed as a new way to predict the success of chemotherapy^[13]^. Ultimately, it might turn out that as cancers evolve and become more drug-resistant, they begin to morph into cells that are more like non-cancer cells in their metabolic pathways and physiology (and hence relatively resistant to chemotherapy) while still maintaining their growth, invasiveness, and metastatic phenotypes.

There are approximately 10,000 articles listed in PubMed that relate to mechanisms of drug resistance in cancer, yet we are still unable to identify the key features that result in the terminal multidrug resistance of many cancers. This conundrum was pointed out over a decade ago by Piet Borst in a prescient commentary in which he coined the term “pan-resistance” to describe this terminal resistance state^[14]^. In this article, he explores a variety of explanations for this phenomenon and concludes that much more work is needed, particularly in the design of relevant animal models to study this phenomenon. We agree that much more information is needed and encourage the submission of articles that explore in more detail the basis of known molecular mechanisms of resistance, identify novel mechanisms of resistance, novel model systems, novel methods of analysis, and novel testable hypotheses.

It is worth considering the ATP-binding cassette (ABC) efflux transporters as a case in point. A substantial part of the existing literature on multidrug resistance is devoted to papers describing new inhibitors of P-glycoprotein (ABCB1) and the ABC transporters ABCG2 (BCRP) and the ABCC family, especially ABCC1 (MRP1). Although these transporters do frequently appear as the cause of multidrug resistance in tissue culture models, and even animal models of multidrug resistance^[15,16]^, their major contribution to drug resistance appears to be related to their contributions to ADMET (absorption, distribution, metabolism, excretion, and toxicity) for anti-cancer drugs and many other drugs, and to their barrier functions, particularly the blood-brain barrier where their expression in capillary endothelial cells in the brain prevents brain accumulation of a high percentage of drugs in common use, including many chemotherapeutics^[17]^. There is a clear role for ABC transporters in some cases of drug resistance in the clinic (Robey *et al*.^[18]^), and new drugs targeted to kill cancer cells frequently turn out to be substrates for ABC transporters^[19]^. However, more often than not, even for drugs known to be substrates for P-glycoprotein or ABCG2 efflux pumps, other mechanisms of resistance supervene. The consideration here for the cancer cell is what is the most efficient way to survive killing by anti-cancer drugs, and the use of ABC transporters is quite energy dependent.

In another very thoughtful commentary, Borst warns against expansive and inaccurate claims made about the clinical significance of expression of the ABC transporters in tumor cells^[20]^. There is ample reason to study the complex mechanism of action, substrate and inhibitor specificity, physiological function, and pharmacologic impact of the ABC transporters, but claiming that P-glycoprotein (and even ABCG2 and ABCC1) is “responsible for multidrug resistance in cancer” and that there are no adequate inhibitors can be misleading without additional data implicating these transporters in specific cancers treated with specific drugs. It is our hope that articles submitted to the Special Collection of *Cancer Drug Resistance* will shed new light on the role of the ABC transporters in multidrug resistance, but not focus on another new inhibitor unless it is truly miraculous!

## SOME QUESTIONS THAT STILL NEED TO BE ANSWERED ABOUT CANCER MULTIDRUG RESISTANCE

Although not intended to be a complete list of challenges remaining in the field of multidrug resistance, there are still quite a few basic questions that need to be more clearly formulated and answered in the literature and would be excellent subjects for papers submitted to this *Cancer Drug Resistance* Special Collection:

(1) Does resistance to a specific chemotherapy protocol reside among the pre-treatment population of cells, and is selected by the therapy, or does it arise through genetic or epigenetic changes in cancer during the course of therapy? This question harkens back to the 1969 Nobel Prize winning work of Luria and Delbruck^[21]^ that demonstrated using a fluctuation analysis that mutations to resistance pre-existed in the pre-treatment population of bacteria. Similar studies in cultured mammalian cells have confirmed these conclusions, and more recent studies on drug resistance in acute myeloid leukemia (AML), a disease where refractory cells often express ABC transporters^[22,23]^ suggest that most resistant subclones that arise were present prior to therapy^[24]^. Similar conclusions were reached using melanoma cells and a lineage mapping approach known as FateMap^[25]^. However, given that most chemotherapy is mutagenic, and that there are quite a few robust transcriptional and epigenetic responses to toxic therapy, it seems unlikely that all cancers will become resistant based on the intrinsic resistance of pre-treatment subclones. This is not an entirely academic question, since if pre-existing mechanisms of resistance can be detected, this would help guide chemotherapy. If resistance occurs post-treatment, then careful monitoring of the tumor population, as by circulating cancer cells or DNA, becomes essential unless these mechanisms turn out to be universal.

(2) Much of the progress in understanding multidrug resistance to date has been based on the application of new technologies. What new technologies should be used to catalog and determine the clinical significance of potential mechanisms of drug resistance? One obvious example is the use of CRISPR-based selections to determine whether increased or decreased expression of specific genes enhances cell survival or leads to drug resistance^[26]^. The downside of this approach is that it is limited to testing one gene at a time, but the advantage is that there is an immediate read-out of individual genes contributing to resistance without the need to identify a gene or genes whose expression or function is altered in tissue culture cells selected for resistance to a specific drug. Another technology that should reveal important information about pre-existing *vs*. induced resistance is lineage tracing, which allows the tracking of individual cells during the drug-selection process^[27]^. The application of both of these approaches to model systems that more closely mimic the growth of human cancers will be an important advance.

(3) Can we develop more tractable model systems to study drug resistance? It is well-established that cultured cancer cells may bear little or no resemblance phenotypically and in terms of gene expression patterns to cells derived directly from cancers^[28]^. Although some characteristics of the tumor of origin may be obtained, the complex networks that confer a selective advantage under adverse conditions are likely altered in cultured cells. So, a variety of different approaches have been taken to study cancer in the laboratory, including genetically engineered mouse models, primary tumor xenografts, usually in mice but more recently in other easier-to-study models such as zebrafish^[29]^, and cancer cell organoids^[30]^. Very few of these models have been employed to study drug resistance, and more detailed studies of the evolution of drug resistance resulting in terminal pan-drug resistance, utilizing new, more informative technologies, would be welcome.

(4) Why do many cancers, after months or years of therapy and periods of dormancy, emerge, grow, and metastasize in a state that is entirely resistant to therapy? This is a problem in clinical research, requiring detailed timelines of cancer evolution enabled by improved molecular imaging, study of circulating cancer cells and DNA, and either repeat biopsies or rapid autopsies to allow transcriptional profiling of terminal cancers. Very few of these studies have been undertaken, but investment of the time and resources needed to do these studies would yield huge returns in knowledge and improved treatment.

## CONCLUSIONS

Cancer is a difficult adversary. The investment in new, more targeted therapeutics has resulted in improved response, but multidrug resistance remains the major barrier to curative treatment of cancer with chemotherapeutics. New advances in technologies to detect drug resistance genes and model systems in which to study the evolution of cancer drug resistance should accelerate discovery in this area. *Cancer Drug Resistance* welcomes manuscripts that address the questions outlined in this Editorial as well as many others that are relevant and give new insights into the problem of multidrug resistance in cancer.

## DECLARATIONS

### Copyright

© The Author(s) 2023.
